# Perioperative Platelet Count Ratio Predicts Long-Term Survival after Left Pancreatectomy and Splenectomy for Pancreatic Adenocarcinoma

**DOI:** 10.3390/jcm13041050

**Published:** 2024-02-12

**Authors:** Ido Nachmany, Hallbera Gudmundsdottir, Hila Meiri, Pavel Eidelman, Ofir Ziv, Lior Bear, Nadav Nevo, Harel Jacoby, Rony Eshkenazy, Ron Pery, Niv Pencovich

**Affiliations:** 1 Department of General Surgery and Transplantation, Sheba Medical Center, Tel Hashomer, Tel-Aviv University, Tel-Aviv 52621, Israel; ido.nachmany@sheba.health.gov.il (I.N.); hila.meiri@sheba.health.gov.il (H.M.); pavel.eidelman@sheba.health.gov.il (P.E.); ofir.ziv@sheba.health.gov.il (O.Z.); lior.bear@sheba.health.gov.il (L.B.); nadav.nevo@sheba.health.gov.il (N.N.); harel.jacoby@sheba.health.gov.il (H.J.); rony.eshkenazy@sheba.health.gov.il (R.E.); ron.pery@sheba.health.gov.il (R.P.); 2 Department of Surgery, Mayo Clinic, Rochester, MN 55905, USA

**Keywords:** thrombocytosis, distal pancreatectomy, biomarker

## Abstract

Background: The value of platelet characteristics as a prognostic factor in patients with pancreatic adenocarcinoma (PDAC) remains unclear. Methods: We assessed the prognostic ability of post-splenectomy thrombocytosis in patients who underwent left pancreatectomy for PDAC. Perioperative platelet count ratio (PPR), defined as the ratio between the maximum platelet count during the first five days following surgery and the preoperative level, was assessed in relation to long-term outcomes in patients who underwent left pancreatectomy for PDAC between November 2008 and October 2022. Results: A comparative cohort of 245 patients who underwent pancreaticoduodenectomy for PDAC was also evaluated. The median PPR among 106 patients who underwent left pancreatectomy was 1.4 (IQR1.1, 1.8). Forty-six had a PPR ≥ 1.5 (median 1.9, IQR1.7, 2.4) and 60 had a PPR < 1.5 (median 1.2, IQR1.0, 1.3). Patients with a PPR ≥ 1.5 had increased median overall survival (OS) compared to patients with a PPR < 1.5 (40 months vs. 20 months, *p* < 0.001). In multivariate analysis, PPR < 1.5 remained a strong predictor of worse OS (HR 2.24, *p* = 0.008). Among patients who underwent pancreaticoduodenectomy, the median PPR was 1.1 (IQR1.0, 1.3), which was significantly lower compared to patients who underwent left pancreatectomy (*p* > 0.001) and did not predict OS. Conclusion: PPR is a biomarker for OS after left pancreatectomy for PDAC. Further studies are warranted to consolidate these findings.

## 1. Introduction

Surgical resection is currently the only curative treatment for PDAC, but unfortunately, only a minority of patients (~20%) are eligible for radical resection due to oncologic and clinical factors [1]. Of those who undergo surgery, nearly half will experience early disease recurrence, within 12 months after surgery, with little benefit of chemotherapy, while the remaining population have longer DFS, prolonged OS, and even cure [2]. To shift towards precision medicine with tight monitoring and early interventions for those at high risk, there is a significant need to identify biomarkers for early recurrence and mortality in patients who have undergone surgical resection.

Platelets have been shown to play a key role in various aspects of cancer development including angiogenesis [3], cell proliferation [4], cell invasiveness, and metastasis [5,6]. Platelets have reciprocal interactions with cancer cells and have been shown to affect cancer progression [7]. At the same time, the presence of a malignant disease affects multiple platelet characteristics and functions [8]. The relationship between platelet counts and pancreatic cancer prognosis is controversial. Some studies have shown that preoperative thrombocytosis in patients with PDAC is associated with a worse long-term outcome, similar to what has been demonstrated in other malignancies such as lung, breast, and colorectal cancer [9,10,11,12,13]. Conversely, other studies have demonstrated improved prognosis in patients with PDAC, with increased platelet counts [14,15]. Recently, it was shown that reduced platelet counts, although still within the normal range, are correlated with advanced tumor stage and worse outcomes in patients with PDAC [16].

The objective of this study was to investigate the association between perioperative platelet counts and long-term survival in patients with PDAC who underwent left pancreatectomy and splenectomy (left pancreatectomy). This particular patient group was chosen due to the unique opportunity provided by the splenectomy part to assess whether post-splenectomy platelet counts, which in healthy patients typically increase due to decreased sequestration and increased thrombogenesis [17], are affected by PDAC, and can predict long-term outcomes. To evaluate this association, a perioperative platelet count ratio (PPR) was calculated, which depicts the ratio between the maximum postoperative platelet count until postoperative day 5 and the preoperative platelet count. PPR was assessed in relation to the long-term outcomes of patients with PDAC who underwent left pancreatectomy. PPR was also evaluated in a parallel cohort of patients with PDAC who underwent pancreaticoduodenectomy without splenectomy.

## 2. Materials and Methods

This retrospective study included patients who underwent left pancreatectomy and pancreaticoduodenectomy for PDAC in a hepato-pancreato-biliary referral center from November 2008 to October 2022. Patients who underwent surgery for non-PDAC lesions, those under the age of 18, and patients who were pregnant at least one year prior to cancer diagnosis were excluded. None of the patients in this cohort had a history of idiopathic thrombocytopenia purpura, essential thrombocythaemia, or any other chronic disease that is known to chronically affect platelet counts. The PPR was calculated by dividing the maximum platelet count up to postoperative day 5 by the preoperative level (the latest blood count taken prior to surgery). All patients in our cohort had at least 4 blood tests with platelet counts during the first 5 days after surgery. Data were extracted retrospectively from the electronic medical charts of the surgery and oncology departments using MDClone© software, a data extraction and synthesis tool connected to the medical records of patients treated in our facility (http://www.mdclone.com, accessed on 1 January 2023). The data collected included patient demographics, medical history, comorbidities, tumor characteristics on imaging, neoadjuvant treatment details, perioperative laboratory indices, surgical pathology, and postoperative course including complications, recurrence, and survival. The collected data were manually assessed and validated for all patients. The rates of missing data among the variables collected are depicted in the Supplementary Table S1. Postoperative complications were classified according to the Clavien–Dindo system, and major complications defined as Clavien–Dindo >3a [18]. ChatGPT was used to improve grammar and context. All methods were carried out in accordance with the Helsinki declaration. The study was approved by the institutional review board at Sheba Medical Center. The informed consent requirement was waived by the ethics committee for this retrospective study with reference committee number SMC-9498-22.

### Statistical Analysis

Continuous variables are presented as median and interquartile range (IQR), and categorical variables as number and percentage. The optimal cutoff point for PPR for predicting OS was determined using the maximally selected rank statistic [19]. Differences between groups were compared using the Mann–Whitney U test for continuous variables and chi-square or Fisher’s exact tests for categorical variables. Patients with missing data values were excluded from calculation of medians/percentages and statistical comparison for the respective variable. OS and DFS from the time of pancreatectomy were estimated using the Kaplan–Meier method and differences between subgroups were assessed using the log-rank test. Patients who died within 60 days from surgery were excluded from survival analysis. Multivariable Cox proportional hazard regression analysis was used to evaluate the effect of PPR on OS and DFS. Two-sided *p*-values of less than 0.05 were considered statistically significant. All statistical calculations were performed using R (version 4.0.0).

## 3. Results

Between November 2008 and October 2022, 106 patients underwent left pancreatectomy for PDAC. The median PPR for the entire cohort was 1.4 (IQR 1.1, 1.8). Using the maximally selected rank statistic for optimal cutoff-point determination, the most significant difference in OS was observed when a PPR cutoff point of 1.5 was used (Supplementary Figure S1). PPR was ≥1.5 in 46 patients (43%) and <1.5 in 60 patients (57%). Table 1 summarizes patient characteristics and preoperative data. No significant differences were observed between the two groups in respect to sex, age, body mass index (BMI), smoking history, and comorbidities. Oncologic characteristics, such as the rates of receiving neoadjuvant therapy and the number of cycles of neoadjuvant therapy given in those who received it, as well as preoperative tumor markers, were similar between the two groups. Among the various laboratory parameters evaluated, the median preoperative platelet count was higher in patients with a PPR < 1.5 than in those with a PPR ≥ 1.5 (195 vs. 169, *p* = 0.002), as was the median platelet to lymphocyte ratio (PLR) (113 vs. 96, *p* = 0.044).

No significant differences were found in preoperative CRP; total white blood cell, neutrophil, or lymphocyte counts; neutrophil to lymphocyte ratio; or hemoglobin–albumin–lymphocyte–platelet score between the two groups (Table 1).

We further investigated whether there were differences in surgery and postoperative course between patients with a PPR ≥ 1.5 compared to those with a PPR < 1.5 (Table 2). No significant differences were found in the rate of surgeries that were performed using a minimally invasive approach, or in median operative time. The rates of surgical complications, reoperation, and readmissions, as well as the average length of hospital stay, were comparable between the two groups. Patients with a PPR ≥ 1.5 had higher maximum CRP (median 210 vs. 173, *p* = 0.035) and lymphocyte count (median 2.7 vs. 2.1, *p* = 0.012) within the first 5 days following surgery. The maximum platelet count during the first 5 days after surgery was significantly higher in patients with a PPR ≥ 1.5 compared to those with a PPR < 1.5 (median 332 vs. 222, *p* < 0.001), even though this group had a significantly lower preoperative platelet count. Final pathology examination was comparable between the two groups in respect to tumor size, rate of R1 resection, number of lymph nodes sampled and lymph nodes positive for malignancy, lymphovascular invasion, and perineural invasion (Table 2). Rates of receiving adjuvant therapy and the number of cycles of adjuvant therapy given in those who received it were comparable between the groups (Table 2).

**Figure 1:**
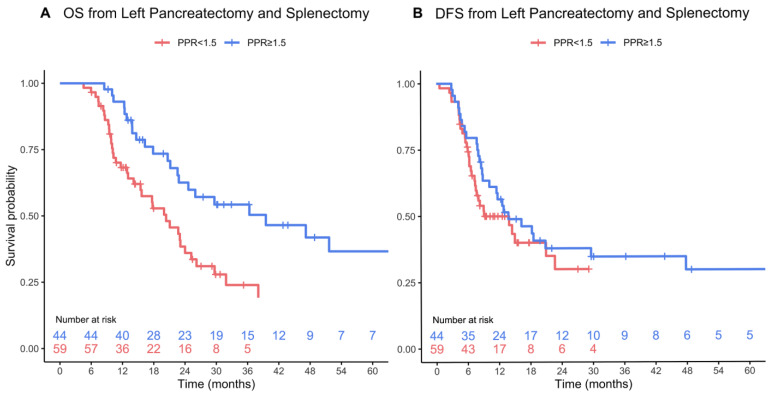
OS (*p* < 0.001) (**A**) and DFS (*p* = 0.21) (**B**) from distal pancreatectomy and splenectomy stratified by PPR. Survival curves are truncated when fewer than five patients remain at risk.

**Figure 2:**
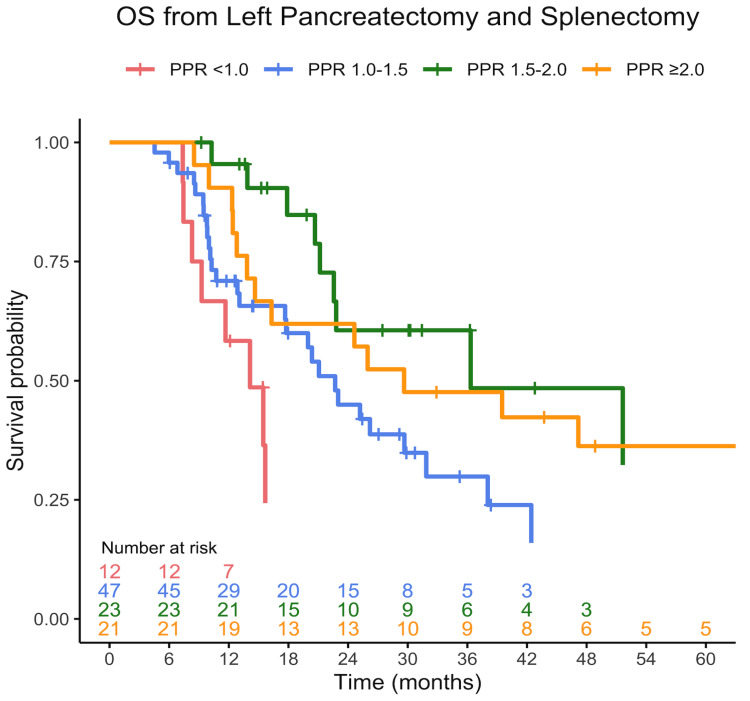
OS from distal pancreatectomy and splenectomy, stratified into distinct groups based on PPR. Patients with PPR < 1 exhibited significantly shorter long-term survival compared to those falling within the PPR range of 1–1.5 (*p* = 0.017). Both the PPR < 1 and PPR 1–1.5 groups had reduced OS compared to patients with PPR ≥ 1.5 (*p* = 0.02). No significant difference was observed between patients with PPR 1.5–2 and those with PPR > 2 (*p* = 0.46). Curves are truncated when fewer than five patients remain at risk.

Patients with a PPR ≥ 1.5 had significantly longer OS following surgery, with a median OS of 40 months (95% CI 23—not reached) compared to 20 months (95% CI 16–26) in patients with a PPR < 1.5 (*p* < 0.001) (Figure 1A). Median DFS was 14 months (95% CI 10-not reached) in patients with a PPR ≥ 1.5, and 9 months (95% CI 8-not reached) in patients with a PPR < 1.5 (*p* = 0.21) (Figure 1B). Among patients with a PPR < 1.5, we observed a distinct gradient in long-term OS based on PPR levels. Specifically, those with the lowest PPR, PPR < 1, experienced significantly worse OS compared to those with PPR in the range of 1–1.5. In contrast, this continuum in OS was not evident among patients with a PPR ≥ 1.5 (Figure 2).

In a multivariable Cox proportional hazard model adjusting for maximal postoperative CRP, lymphocyte, and platelet counts (preoperative and maximal postoperative levels), a PPR < 1.5 significantly correlated with worse OS (hazard ratio—2.24 (95% CI 1.23–4.06, *p* = 0.008)) (Table 3). At the end of the study period, 41 patients remained alive, with a median follow-up of 27 months (IQR 14, 38).

**Figure 3:**
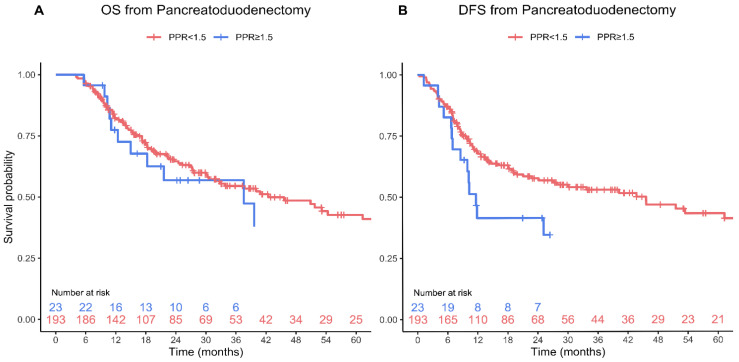
OS (*p* = 0.59) (**A**) and DFS (*p* = 0.11) (**B**) from pancreatoduodenectomy stratified by PPR. Survival curves are truncated when fewer than five patients remain at risk.

To explore the potential impact of the splenectomy on the observed correlation between PPR and outcomes in patients with PDAC that underwent left pancreatectomy, we analyzed a comparative cohort of 245 PDAC patients who underwent pancreaticoduodenectomy, which does not include a splenectomy. The median PPR in this group was 1.1 (IQR 0.9, 1.3), which was significantly lower than the median PPR in the left pancreatectomy group (*p* < 0.001). Using the same cutoff as for left pancreatectomy, 28 patients (11%) were identified with a PPR > 1.5 and 217 patients (89%) with a PPR < 1.5. Patients with a PPR > 1.5 had increased rates of chronic renal failure (18% vs. 5.5%, *p* = 0.032), a lower preoperative platelet count (median 176 vs. 246, *p* < 0.001) and lymphocyte count (median 1.2 vs. 1.7, *p* = 0.012), and were more likely to have undergone neoadjuvant therapy (43% vs. 21%, *p* = 0.021). No other differences were found between the two groups with respect to patient demographics, medical history, comorbidities, or oncologic data (Table 4). Similarly, no major differences were observed in the data pertaining to surgery or the postoperative course between the two groups (Table 5). Notably, patients with high PPR in the pancreaticoduodenectomy group had comparable long-term outcomes to those with low PPR (Figure 3), and no cutoff value for PPR with a significant effect on OS was identified.

## 4. Discussion

In this study, we conducted a retrospective examination of the potential role of PPR as a novel biomarker for long-term outcomes in patients with PDAC who underwent curative resection. Our findings demonstrate that PPR is a significant predictor of OS in patients with PDAC who underwent left pancreatectomy. Patients with PPR ≥ 1.5 had significantly longer survival after surgery compared to those with PPR < 1.5. Notably, PPR was not found to be predictive in patients who underwent pancreaticoduodenectomy without splenectomy.

Post-splenectomy reactive thrombocytosis is a well-known phenomenon affecting over 80% of patients undergoing splenectomy, with platelet counts usually increasing by 30–100% within days after surgery [20,21,22]. Pro-platelets are released into the blood stream from megakaryocytes within the bone marrow, mainly under the regulation of thrombopoietin, and mature as platelets in the circulation. Normally, up to one-third of the total platelet mass is sequestered in the spleen as an interchangeable pool [17]. Studies have shown that surgical trauma and the associated inflammatory response led to increased ploidy of bone marrow megakaryocytes resulting in increased thrombopoiesis. Adding a splenectomy further augments this phenomenon [23]. Therefore, it is assumed that post-splenectomy thrombocytosis is attributed to both the elimination of splenic sequestration and increased bone marrow production.

The association between the platelet counts and outcomes of patients with PDAC is conflicting. While some studies have demonstrated that thrombocytosis is associated with unfavorable outcomes [24,25,26], other studies shown the opposite, or no association at all [27,28]. Although most studies have used a single measurement of baseline platelets, usually pretreatment, one study has shown that time-varying post-diagnosis thrombocytopenia is associated with poor survival of patients with PDAC [15]. Tumor-induced alterations in bone marrow myelopoiesis and thrombopoiesis are driven by growth factors and cytokines secreted by the primary tumor, as well as by other mechanisms [29,30,31]. Nevertheless, the overall effect of cancer on peripheral blood platelet counts can be masked by factors such as alterations in splenic sequestration [17].

We suggest that in patients with PDAC that undergo left pancreatectomy (with splenectomy), the splenectomy acts as a “stress test” for the thrombopoietic ability of the patient, in addition to eliminating the effect of splenic sequestration. PPR represents the ‘ability’ of the patient to increase platelet counts after the major surgery and splenectomy. This ability may be related to the extent to which the patient’s bone marrow is affected by the tumor, which may reflect both tumor and patient status. Although delineating the mechanism of this phenomenon is beyond the scope of this report, it is evident that patients in whom the postoperative platelet count was at least 1.5-fold higher than the preoperative levels had significantly improved long-term outcomes compared to those with a PPR < 1.5, presumably due to favorable yet-to-be-identified patient or tumor characteristics. Notably, patients with a PPR < 1 had the worse outcomes, further supporting the concept that PPR may represent a continuum of unfavorable to favorable patient outcomes. Importantly, no significant differences were noted between the groups in respect to common preoperative patients or tumor characteristics, including the rate of receiving neoadjuvant treatment, which may affect platelet counts. Assessment of the maximal postoperative platelet count within the first five days after surgery to calculate PPR provides sufficient time for thrombocytosis to occur and is practical for clinical use, as laboratory tests are commonly performed during this period for most patients. Using a single test, the maximal platelet count for PPR calculation may be criticized for not accurately reflecting the patient’s thrombopoietic ability, given that this value can be affected by multiple factors, including fluid status and others. However, there were no cases in this cohort in which the maximal count was disproportionally higher than neighboring tests. It is noteworthy that other formulas, such as those incorporating a combination of several postoperative platelet count measurements, including average platelet counts, were less predictive of long-term outcomes. PPR using POD7, which was the median length of stay in our cohort, yielded the same results as POD5. We believe that using POD5 is superior since more patients are still hospitalized at that time point after surgery. Postoperative complications may affect the patient’s inflammatory status and influence platelet counts. However, no significant differences in minor or major postoperative complications were observed between patients with a PPR ≥ 1.5 and those with a PPR < 1.5, including infectious complications. These findings are in good agreement with our data, demonstrating no differences in receiving adjuvant chemotherapy between the groups. Notably, a PPR ≥ 1.5 was not associated with favorable pathology parameters such as RO/R1 resection or lymph nodes involvement. Maximum postoperative CRP and lymphocyte counts were higher in patients with a PPR ≥ 1.5 but did not affect the relationship between PPR and OS in multivariate analysis. Overall, this may suggest that PPR may reflect a combination of parameters, rather than just identifiable tumor factors and inflammatory status. Although the DFS of patients with a PPR < 1.5 was 5 months shorter than those with a PPR ≥ 1.5, this difference did not reach statistical significance. We believe that this is mainly due to insufficient cohort size. However, this is also remains to be proven in a larger study. The observation that PPR was not predictive in patients who underwent pancreaticoduodenectomy highlights the role of splenectomy in unmasking the thrombopoietic ability of patients with PDAC. Hence, it is plausible that PPR may also predict long-term outcomes in other patients with cancer who undergo surgeries involving splenectomy.

The limitations of this study include its retrospective design, and the fact that it was conducted at a single center. Moreover, we acknowledge that the relatively small cohort size of 106 patients in our study represents a significant drawback. This constraint might limit the generalizability of our results, as the findings derived from a smaller sample may not accurately reflect broader patterns and trends. Additionally, the modest cohort size could affect the statistical power of our analyses, potentially hindering our ability to detect more nuanced effects or associations. Therefore, we view our results as a proof of concept. We recognize the importance of further validation and encourage other research groups to corroborate these findings, ideally through larger-scale, prospective studies. Such subsequent research efforts are essential to confirm and expand upon our initial observations. The maximal platelet count after surgery may be influenced by different protocols for blood tests in various centers. In our cohort, all patients had at least four blood tests during the first five days after surgery; this might not be the case in other centers. However, since post-splenectomy thrombocytosis typically persists for several weeks to months after surgery, we believe that platelet counts obtained during clinic visits after surgery may be used to calculate PPR with similar predictive ability. However, further studies are needed to confirm this hypothesis.

## 5. Conclusions

This study identifies PPR as a novel and powerful biomarker for long-term outcomes in patients with PDAC who undergo left pancreatectomy with splenectomy. PPR is simple to obtain and can easily identify patients with a worse prognosis who may benefit from aggressive follow-up and adjuvant treatments. A PPR of 1.5 was found to be an appropriate and convenient cutoff point for OS. Additional studies are necessary to confirm and extend these findings, as well as to determine the generalizability of PPR to other patient populations.

## Figures and Tables

**Table 1:** Patient characteristics and preoperative data among patients who underwent left pancreatectomy and splenectomy.

	All Patients n = 106	PPR ≥ 1.5 n = 46	PPR < 1.5 n = 60	*p*
Female sex, n (%)	51 (49)	24 (53)	27 (45)	0.52
Age, median (IQR), years	68 (61, 75)	67 (61, 72)	70 (60, 75)	0.17
BMI, median (IQR), kg/m^2^	25.2 (23.1, 28.5)	25.2 (23.3, 28.1)	25.2 (22.8, 28.8)	0.90
Smoking, n (%)	19 (19)	8 (18)	11 (19)	0.99
Hypertension, n (%)	42 (40)	15 (33)	27 (45)	0.27
Ischemic heart disease, n (%)	7 (6.6)	3 (6.5)	4 (6.7)	0.99
Congestive heart failure, n (%)	1 (0.9)	0 (0.0)	1 (1.7)	0.99
Diabetes, n (%)	31 (29)	9 (20)	22 (37)	0.09
Chronic obstructive pulmonary disease, n (%)	5 (4.7)	2 (4.3)	3 (5.0)	0.99
Asthma, n (%)	5 (4.7)	2 (4.3)	3 (5.0)	0.99
Chronic renal failure, n (%)	4 (3.8)	0 (0.0)	4 (6.7)	0.13
Neoadjuvant therapy, n (%)	32 (30)	14 (30)	18 (30)	0.99
Preoperative CA 19-9, median (IQR), U/mL	41 (18, 110)	32 (20, 90)	65 (18, 175)	0.26
Preoperative CEA, median (IQR), µg/L	2.8 (1.7, 4.5)	2.8 (1.9, 4.2)	2.7 (1.6, 4.5)	0.95
Preoperative albumin, median (IQR) g/dL	3.8 (3.3, 4.2)	3.7 (3.2, 4.1)	3.8 (3.4, 4.2)	0.24
Preoperative bilirubin, median (IQR), mg/dL	0.5 (0.4, 0.6)	0.5 (0.4, 0.7)	0.5 (0.4, 0.6)	0.16
Preoperative CRP, median (IQR), mg/L	4.9 (1.9, 15.6)	5.6 (1.9, 25.6)	4.1 (1.9, 11.0)	0.77
Preoperative WBC count, median (IQR), 10^9^/L	7.1 (5.8, 9.2)	7.6 (5.8, 11.2)	6.8 (5.8, 8.6)	0.35
Preoperative neutrophil count, median (IQR), 10^9^/L	4.3 (3.4, 6.1)	4.3 (3.3, 6.7)	4.3 (3.4, 5.5)	0.82
Preoperative lymphocyte count, median (IQR), 10^9^/L	1.7 (1.3, 2.2)	1.7 (1.2, 2.2)	1.8 (1.3, 2.2)	0.73
Preoperative platelet count median (IQR), 10^9^/L	181 (149, 216)	169 (137, 197)	195 (157, 226)	0.002
Preoperative Hb, median (IQR), g/dL	12.8 (11.5, 13.6)	12.8 (11.5, 13.6)	12.9 (11.6, 13.7)	0.78
PLR, median (IQR)	106 (85, 144)	96 (75, 135)	113 (94, 155)	0.044
NLR, median (IQR)	2.4 (1.8, 3.8)	2.1 (1.8, 4.4)	2.5 (1.8, 3.2)	0.99
HALP score, median (IQR)	43 (28, 57)	43 (28, 72)	43 (31, 52)	0.62

PPR—perioperative platelet ratio; IQR—interquartile range; BMI—body mass index; WBC—white blood cell; PLR—platelet to lymphocyte ratio; NLR—neutrophil to lymphocyte ratio; HALP, hemoglobin-albumin-lymphocyte-platelet.

**Table 2:** Surgery, postoperative course, and pathology among patients who underwent left pancreatectomy and splenectomy.

	All Patients n = 106	PPR ≥ 1.5 n = 46	PPR < 1.5 n = 60	*p*
Laparoscopic approach, n (%)	64 (60)	31 (67)	33 (55)	0.27
Operative time, median (IQR), min	290 (215, 372)	266 (205, 345)	302 (221, 380)	0.23
Major complications (CD ≥ 3), n (%)	20 (19)	5 (11)	15 (25)	0.11
Life threatening complication (CD ≥ 3) ^#^, n (%)	2 (1.9)	1 (2.2)	1 (1.6)	0.337
Postpancreatectomy hemorrhage, n (%) ^$^	3 (2.83)	1 (2.2)	2 (3.3)	0.153
Reoperation, n (%)	6 (5.6)	2 (4.3)	4 (6.6)	0.16
Length of stay, median (IQR), days	7.0 (5.0, 10.0)	7.0 (5.0, 9.0)	7.0 (6.0, 10.5)	0.13
Readmission, n (%)	31 (29)	11 (24)	20 (33)	0.40
Postoperative CRP, median (IQR), mg/L ^a^	194 (146, 244)	210 (172, 248)	173 (137, 216)	0.035
Postoperative lymphocyte count, median (IQR), 10^9^/L ^a^	2.4 (1.7, 3.2)	2.7 (2.0, 3.8)	2.1 (1.6, 2.8)	0.012
Postoperative platelets, median (IQR), 10^9^/L ^a^	258 (204, 338)	332 (264, 439)	222 (197, 268)	<0.001
PPR, median (IQR)	1.4 (1.1, 1.8)	1.9 (1.7, 2.4)	1.2 (1.0, 1.3)	<0.001
Adjuvant therapy, n (%)	64 (62)	30 (67)	34 (59)	0.53
No. of cycles, median (IQR)	4.5 (3.0, 8.0)	4.5 (3.0, 7.0)	4.5 (3.0, 8.0)	0.78
Tumor size, median (IQR), cm	3.1 (2.3, 4.2)	3.1 (2.5, 4.0)	3.0 (2.0, 4.3)	0.92
R1 resection, n (%)	16 (17)	9 (23)	7 (13)	0.39
No. of sampled lymph nodes, median (IQR)	13 (9, 17)	12 (8, 17)	14 (10, 17)	0.31
Lymph node involvement, n (%)	41 (45)	16 (40)	25 (49)	0.52
No. of positive lymph nodes, median (IQR)	0 (0, 2)	0 (0, 2)	0 (0, 1)	0.68
Lymphovascular invasion, n (%)	16 (18)	9 (24)	7 (14)	0.34
Perineural invasion, n (%)	54 (61)	24 (63)	30 (60)	0.94

Abbreviations: PPR, perioperative platelet ratio; IQR, interquartile range; CD, Clavien–Dindo. ^a^ Maximum level during postoperative days 1–5. ^#^ requiring intensive care unit management. ^$^ requiring intervention under local of general anesthesia.

**Table 3:** Multivariable Cox proportional hazard regression analysis including postoperative parameters that had *p* < 0.05 in univariate analysis.

	Univariate HR (95% CI)	*p*-Value	Multivariate HR (95% CI)	*p*-Value
PPR < 1.5 ^#^	2.47 (1.43–4.26)	0.001	2.24 (1.23–4.06)	0.008
Platelet count *	0.998 (0.996–0.999)	0.037	1.0011 (0.997–1.001)	0.24
CRP *	0.999 (0.997–1.001)	0.42	0.997 (0.9973–1.002)	0.79
Lymphocyte count *	0.95 (0.78–1.15)	0.58	1.07 (0.88–1.30)	0.52

^#^ Separate multivariate analysis using preoperative platelet counts yielded HR of 2.46 (95%CI 1.33–4.56). * Maximal levels within 5 days from surgery.

**Table 4:** Patient characteristics and preoperative data among patients who underwent pancreaticoduodenectomy.

	All Patients n = 245	PPR ≥ 1.5 n = 28	PPR < 1.5 n = 217	*p*
Female sex, n (%)	119 (49)	14 (50)	105 (48)	0.99
Age, median (IQR), years	70 (63, 75)	65 (57, 76)	70 (63, 75)	0.17
BMI, median (IQR), kg/m^2^	26.0 (23.7, 29.8)	27.5 (24.2, 31.5)	25.6 (23.6, 29.3)	0.19
Smoking, n (%)	76 (31)	11 (39)	65 (30)	0.43
Hypertension, n (%)	121 (49)	16 (57)	105 (48)	0.50
Ischemic heart disease, n (%)	23 (9.4)	4 (14)	19 (8.8)	0.31
Congestive heart failure, n (%)	5 (2.0)	0 (0.0)	5 (2.3)	0.99
Diabetes, n (%)	96 (39)	10 (36)	86 (40)	0.85
Chronic obstructive pulmonary disease, n (%)	5 (2.0)	0 (0.0)	5 (2.3)	0.99
Asthma, n (%)	17 (6.9)	1 (3.6)	16 (7.4)	0.70
Chronic renal failure, n (%)	17 (6.9)	5 (18)	12 (5.5)	0.032
Neoadjuvant therapy, n (%)	58 (24)	12 (43)	46 (21)	0.021
Preoperative CA 19-9, median (IQR), U/mL	161 (54, 523)	159 (66, 590)	163 (54, 466)	0.88
Preoperative CEA, median (IQR), µg/L	2.8 (2.0, 4.7)	2.6 (1.7, 4.4)	2.9 (2.0, 4.8)	0.33
Preoperative albumin, median (IQR) g/dL	3.8 (3.3, 4.2)	3.7 (3.4, 4.2)	3.8 (3.3, 4.2)	0.97
Preoperative bilirubin, median (IQR), mg/dL	1.7 (0.6, 7.8)	0.9 (0.5, 5.0)	2.0 (0.6, 7.8)	0.20
Preoperative CRP, median (IQR), mg/L	6.6 (2.2, 15.4)	5.7 (2.0, 12.3)	7.1 (2.2, 15.5)	0.52
Preoperative WBC count, median (IQR), 10^9^/L	7.2 (6.1, 9.1)	6.3 (4.8, 8.5)	7.2 (6.1, 9.1)	0.14
Preoperative lymphocyte count, median (IQR), 10^9^/L	1.6 (1.2, 2.2)	1.2 (0.8, 1.8)	1.7 (1.3, 2.2)	0.012
Preoperative platelet count, median (IQR), 10^9^/L	239 (195, 296)	176 (134, 222)	246 (202, 303)	<0.001
Preoperative Hb, median (IQR), g/dL	12.0 (11.2, 13.0)	11.6 (10.9, 12.6)	12.1 (11.3, 13.0)	0.20

PPR—perioperative platelet ratio; IQR—interquartile range; BMI—body mass index; WBC—white blood cell.

**Table 5:** Surgery, postoperative course, and pathology among patients who underwent pancreaticoduodenectomy.

	All Patients n = 245	PPR ≥ 1.5 n = 28	PPR < 1.5 n = 217	*p*
Laparoscopic approach, n (%)	33 (13)	5 (18)	28 (13)	0.67
Operative time, median (IQR), min	378 (327, 444)	366 (341, 451)	378 (317, 444)	0.57
Major complications (CD ≥ 3), n (%)	60 (24)	5 (18)	55 (25)	0.53
Reoperation, n (%)	38 (16)	4 (14)	34 (16)	0.99
Length of stay, median (IQR), days	16 (10, 27)	17 (10, 29)	16 (10, 26)	0.48
Readmission, n (%)	105 (43)	7 (25)	98 (45)	0.07
Postoperative CRP, median (IQR), mg/L ^a^	196 (135, 266)	183 (140, 248)	198 (135, 267)	0.58
Postoperative lymphocyte count, median (IQR), 10^9^/L ^a^	1.7 (1.2, 2.1)	1.5 (1.1, 1.8)	1.7 (1.2, 2.2)	0.16
Postoperative platelets, median (IQR), 10^9^/L ^a^	263 (213, 323)	314 (247, 375)	262 (209, 313)	0.015
PPR, median (IQR)	1.1 (0.9, 1.3)	1.7 (1.6, 1.9)	1.0 (0.9, 1.2)	<0.001
Adjuvant therapy, n (%)	157 (64)	18 (64)	139 (64)	0.99
Tumor size, median (IQR), cm	2.5 (2.0, 3.5)	3.0 (1.7, 3.7)	2.5 (2.0, 3.5)	0.49
R1 resection, n (%)	67 (28)	9 (36)	58 (27)	0.50
No. of sampled lymph nodes, median (IQR)	18 (13, 23)	20 (13, 24)	18 (13, 23)	0.56
Lymph node involvement, n (%)	157 (65)	19 (73)	138 (64)	0.51
No. of positive lymph nodes, median (IQR)	1 (0, 3)	3 (0, 4)	1 (0, 3)	0.14
Lymphovascular invasion, n (%)	71 (31)	5 (20)	66 (33)	0.29
Perineural invasion, n (%)	147 (65)	15 (60)	132 (66)	0.74

PPR—perioperative platelet ratio; IQR—interquartile range; CD—Clavien–Dindo ^a^ Maximum level during postoperative days 1–5.

## Data Availability

The data are available upon request from the corresponding author.

## Supplementary Materials

The following supporting information can be downloaded at: https://www.mdpi.com/article/10.3390/jcm13041050/s1, Table S1. Rate of missing data among the variables collected. Figure S1: The optimal cutpoint for perioperative platelet ratio in predicting overall survival was determined using the maximally selected rank statistic.
