# Supplementary material for: Perioperative Platelet Count Ratio Predicts Long-Term Survival after Left Pancreatectomy and Splenectomy for Pancreatic Adenocarcinoma
Source: J Clin Med. 2024 Feb 12;13(4):1050. doi: 10.3390/jcm13041050 (PMC10888544; doi:10.3390/jcm13041050)
Supplement: Supplementary file 1 [file jcm-13-01050-s001.zip › jcm-2862682-supplementary.pdf]

Supplementary Figure S1: The optimal cutpoint for perioperative platelet ratio in predicting overall survival was determined using the maximally selected rank statistic.

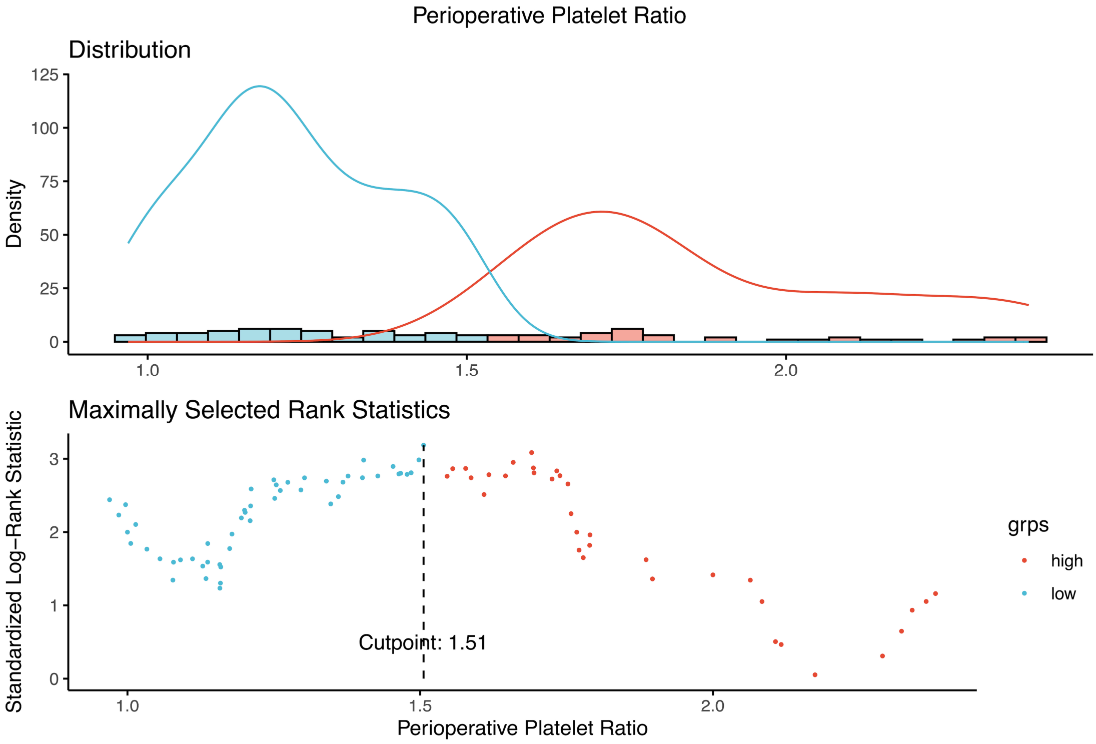

Supplementary Table S1. Rate of missing data among the variables collected.

	Left Pancreatectomy and Splenectomy (n=106)	Pancreaticoduodenectomy (n=245)
Sex	1 (0.9%)	0 (0.0%)
Age	0 (0.0%)	0 (0.0%)
BMI	6 (5.7%)	18 (7.3%)
Smoking	5 (4.7%)	0 (0.0%)
Hypertension	0 (0.0%)	0 (0.0%)
Ischemic heart disease	0 (0.0%)	0 (0.0%)
Congestive heart failure	0 (0.0%)	0 (0.0%)
Diabetes	0 (0.0%)	0 (0.0%)
COPD	0 (0.0%)	0 (0.0%)
Asthma	0 (0.0%)	0 (0.0%)
Chronic renal failure	0 (0.0%)	0 (0.0%)
Neoadjuvant therapy	0 (0.0%)	0 (0.0%)
Preoperative CA 19-9	21 (20%)	67 (27%)
Preoperative CEA	20 (19%)	60 (24%)
Preoperative albumin	5 (4.7%)	17 (6.9%)
Preoperative bilirubin	10 (9.4%)	0 (0.0%)
Preoperative CRP	28 (26%)	55 (22%)
Preoperative WBC count	0 (0.0%)	23 (9.4%)
Preoperative neutrophil count	10 (9.4%)	20 (8.2%)
Preoperative lymphocyte count	10 (9.4%)	20 (8.2%)
Preoperative platelet count	0 (0.0%)	0 (0.0%)
Preoperative Hb	0 (0.0%)	0 (0.0%)
Preoperative PLR	10 (9.4%)	20 (8.2%)
Preoperative NLR	10 (9.4%)	20 (8.2%)
Preoperative HALP score	14 (13%)	22 (8.97%)
Laparoscopic vs. open	0 (0.0%)	0 (0.0%)
Operative time	12 (11%)	59 (24%)
Major complications (CD ≥3)	0 (0.0%)	0 (0.0%)
Reoperation	3 (2.8%)	0 (0.0%)
Length of stay	2 (1.9%)	2 (0.8%)
Readmission	0 (0.0%)	0 (0.0%)
Postoperative CRP	22 (21%)	43 (18%)
Postoperative lymphocyte count	0 (0.0%)	5 (2.0%)
Postoperative platelet count	0 (0.0%)	0 (0.0%)
PPR	0 (0.0%)	0 (0.0%)
Adjuvant therapy	3 (2.8%)	0 (0.0%)
Tumor size	14 (13%)	21 (8.6%)
Margins (R0 vs. R1)	14 (13%)	8 (3.3%)
No. of sampled lymph nodes	15 (14%)	5 (2.0%)
Lymph node involvement	15 (14%)	5 (2.0%)
No. of positive lymph nodes	15 (14%)	5 (2.0%)
Lymphovascular invasion	19 (18%)	18 (7.3%)
Perineural invasion	0 (0.0%)	19 (7.8%)

Abbreviations: BMI, body mass index; COPD, chronic obstructive pulmonary disease; WBC, white blood cell; PLR, platelet to lymphocyte ratio; NLR, neutrophil to lymphocyte ratio; HALP, hemoglobin albumin lymphocyte platelet; PPR, perioperative platelet ratio; CD, Clavien-Dindo.
